# Effects on schooling function in mackerel of sub-lethal capture related stressors: Crowding and hypoxia

**DOI:** 10.1371/journal.pone.0190259

**Published:** 2017-12-28

**Authors:** Nils Olav Handegard, Maria Tenningen, Kirsten Howarth, Neil Anders, Guillaume Rieucau, Michael Breen

**Affiliations:** 1 Marine Ecosystem Acoustics, Institute of Marine Research, Bergen, Norway; 2 Fish Capture Division, Institute of Marine Research, Bergen, Norway; 3 Department of Biology, University of Bergen, Bergen, Norway; Maurice Lamontagne Institute, CANADA

## Abstract

The selectivity of fishing gears with respect to fish species and size is important, both for fisheries management and fishing operations. Purse seining is an efficient, environmentally friendly fish capture methodology generally targeting single species aggregations, but once a fish school has been selected and surrounded by the seine, there is no selections for individual size, species or catch quantity. A common practice for evaluating the catch is to haul the seine to a point where physical samples or inspections of catch composition can be made. The release process is called slipping and may lead to mortality in the released fish. The objective of this study was to simulate a crowding situation and investigate how the behaviour was affected in response to increased fish density, decreased oxygen levels, or a combination of the two, and to see if there is a behavioural measure that can be used to set safe crowding limits. The experiment was conducted on Mackerel (*Scomber scombrus*) held in net pens. The volume of the net pen was reduced to increase fish density, and a tarpaulin bag was wrapped around the pen to reduce the oxygen levels. Oxygen, fish density and space occupancy was monitored during the experiment, and the behavioural reactions was assessed using an imaging sonar. The main result was that the schooling function, i.e. the response to a predator model, was significantly reduced during crowding but not in response to hypoxia. There were some indications of a slow recovery of the function post-treatment. We conclude that crowding causes behavioural responses that occur before densities that induce fish mortality. Consequently, there is a behavioural response that could be used as a proxy for setting safe crowding limits.

## Introduction

Purse seining accounts for about 30% of the world’s total fisheries catches [1], is the most efficient method for catching aggregated pelagic species [2], and is one of the most energy-efficient fishing methods worldwide [3]. In fisheries regulated by catch quotas and minimum landing sizes, the selectivity of the fishing gear is an important aspect, but no accurate tools are currently available for early catch characterization with respect to individual size or quantity in purse seine fisheries [4]. School size and species are evaluated before setting the seine usually with hydro acoustic instruments (sonars and echo-sounders). Accurate pre-catch evaluation can be challenging, especially if fish form large dense aggregations, and may lead to catches mixed with non-target species or large catches that exceed the quota or the loading capacity of the vessel [5,6]. No commercial tools are available for estimating individual size or quality (e.g. fat and stomach contents) pre- and early-catch, both affecting catch value. Catch size and quality, and at times quantity and species, are not possible to evaluate before the catches are crowded by the vessel side and visual and physical samples can be obtained. To avoid burst nets and vessel overloading, and to promote catch value, proportions of the catch may be released. This process of hauling the net, assessing and then releasing the fish is called "slipping", and is achieved by lowering the float line or by making an opening in the bunt end of the net, and allowing the fish to swim out.

The slipping process is not without challenges. If the slipping is performed at the later stages of the operation, the fish may be crowded to a level that can induce significant mortality; more than 80% mortality in mackerel [7] and up 50% in herring [8] have been reported. This indirect fishing mortality may have implications on fisheries management since it may bias stock assessment [9,10], contribute to changes in ecosystem structure and function [11], have fish welfare implications, and waste a valuable protein source for human consumption. This has led to legislation both in Norway [12] and the European Union [13] where discarding of catch (including slipping) is prohibited unless it can be demonstrated that the released fish are not “dying” or have a high likelihood of survival, for the Norwegian and European Union regulations, respectively. To promote responsible fishing operations, there is a need to understand what causes the mortalities during slipping.

The causal explanation for stress and mortalities during slipping is unknown. A typical purse seine catch operation in northern Europe takes about one hour after the net has been set, and in the initial phase, the fish can move freely inside the purse seine [14,15]. As the seine is hauled, the space will be increasingly restricted to a point where school density is too high for free swimming [16]. At the same time oxygen is likely to decrease inside the school, with the depletion rate related to catch size and water flow through the seine. Oxygen saturation has been observed to drop to about 50% following 10 minutes crowding at fish densities above 400 kg m^-3^ (mortality >50%) in controlled net pen experiments with Atlantic herring [8]. Experiments have shown that mortality is related to both the density and duration of crowding, and that careful slipping before high densities are reached results in very low mortalities [8,17,18]. However, the mechanism leading to mortality is unknown, whether it is caused by physical contact, stress or hypoxia.

Atlantic mackerel is an obligatory schooling fish, and schooling is an important and general mechanism to achieve a suite of key functions including feeding, survival and reproduction. These mechanisms include efficient gradient search [19], decision making [20], increased swimming efficiency [21], predator avoidance and information transfer [22,23], among others. Laboratory studies indicate that purse seine fishing and slipping may result in temporary behavioural impairment that could increase susceptibility to predation [24]. It has also been shown that hypoxia, one of the likely key stressors during purse seining, can have detrimental effects on anti-predatory behaviour of schooling species with demonstrated drastic changes in schooling tendency (e.g. reduced school cohesion and coordinated collective swimming) and a lower responsiveness to predators [24,25]. Fish utilizing collective behaviour as a predator avoidance strategy often respond to potential threats by altering their collective responses [26], and in some cases these reactions are altered after being exposed to potential threats. It has been shown that killer whale vocalizations of herring [27] change the reactions to a predator model without the state change being explicitly detected by typical schooling parameters like swimming speed, internal correlation structures and rotational order. The changes in internal state also lead to changes in how the internal signalling within the school occurs [28]. It is likely that the fish behaviour within the school will be affected before the crowding levels that lead to mortalities are reached. If we can detect this shift in behaviour, we could use these behavioural changes as early warning indicators for slipping related stresses, and potentially avoid any related mortality.

The objective of this experiment was to find behavioural indicators of capture related stressors (crowding and hypoxia) at sub-lethal levels, with the aim of identifying early warning indicators to avoid slipping induced mortality in purse seine fishing. This was achieved by monitoring the schooling behaviour in wild-caught mackerel held in aquaculture pens, under simulated crowding and hypoxic conditions. In addition, fish were exposed to a predator model to test if there were changes in their collective state that are not directly observable.

## Materials and methods

To address the objectives, we chose to do the experiment in a net pen allowing for sufficient number of individuals and reasonable amount of control of the stressors. Mackerel was used as a model species since 1) it is an obligatory schooling fish that can form dense schools with the capability to perform highly synchronized collective responses, and 2) has been reported to be vulnerable to crowding [7].

### Pilot experiment and animal welfare

A pilot experiment was conducted at the Institute of Marine Research aquarium facilities, Austevoll, to establish sub-lethal limits for crowding and depleted dissolved oxygen concentrations (hypoxia) during the experiment; under the authority of the Norwegian Animal Research Authority (Mattilsynet) (permission FOTS 7601). Small replicate groups (n ~30) were exposed to a range of hypoxic (>95% to 40% oxygen saturation) and crowding (5 to 51.5 kg.m^-3^) to determine whether these stressors would induce a mortality significantly greater than baseline rates. Following these experiments acceptable stressor limits of crowding (<30 kg.m^-3^) and hypoxia (>40% saturation) were defined and permission given to conduct the main experiment (permission FOTS 7671). During both experiments, the fish were monitored continuously and any individuals that exhibited signs of significant injury or distress were removed. The removed fish were terminated using an overdose of MS222 in a concentration of 500 mg/l or a fatal blow to the head (if the fish was acutely distressed and the MS222 was not readily available).

### Fish capture, fish transfer and maintenance

The fish were captured without any active fishing operation. The side of a standard aquaculture brown-coloured net-pen (12 m long x 12 m wide x 12 m deep) was simply lowered at night, and aquaculture feed was made available within the pen resulting in mackerel moving voluntarily inside. In the morning, the pen was closed and the trapped mackerel was transferred into a holding pen. This avoided fish injuries that is commonly incurred during commercial fishing operations. Approximately 2 tonnes were caught in autumn 2014 and kept in the holding pen over the winter, whereas an additional 5 tonnes were caught in the summer and autumn of 2016. Fish were fed with standard small-sized aquaculture pellets in addition to any naturally available food items that passed into the pen. The mackerel total body length and weight were measured on individuals caught from the school using a landing net (n = 170; body length = 40.5 ± 2.5 cm; weight = 887 ± 161 g; index of fish condition (1 000 x weight x body length^3^) = 13.3 ± 1.4; all results are expressed as a mean ± SD).

**Fig 1:**
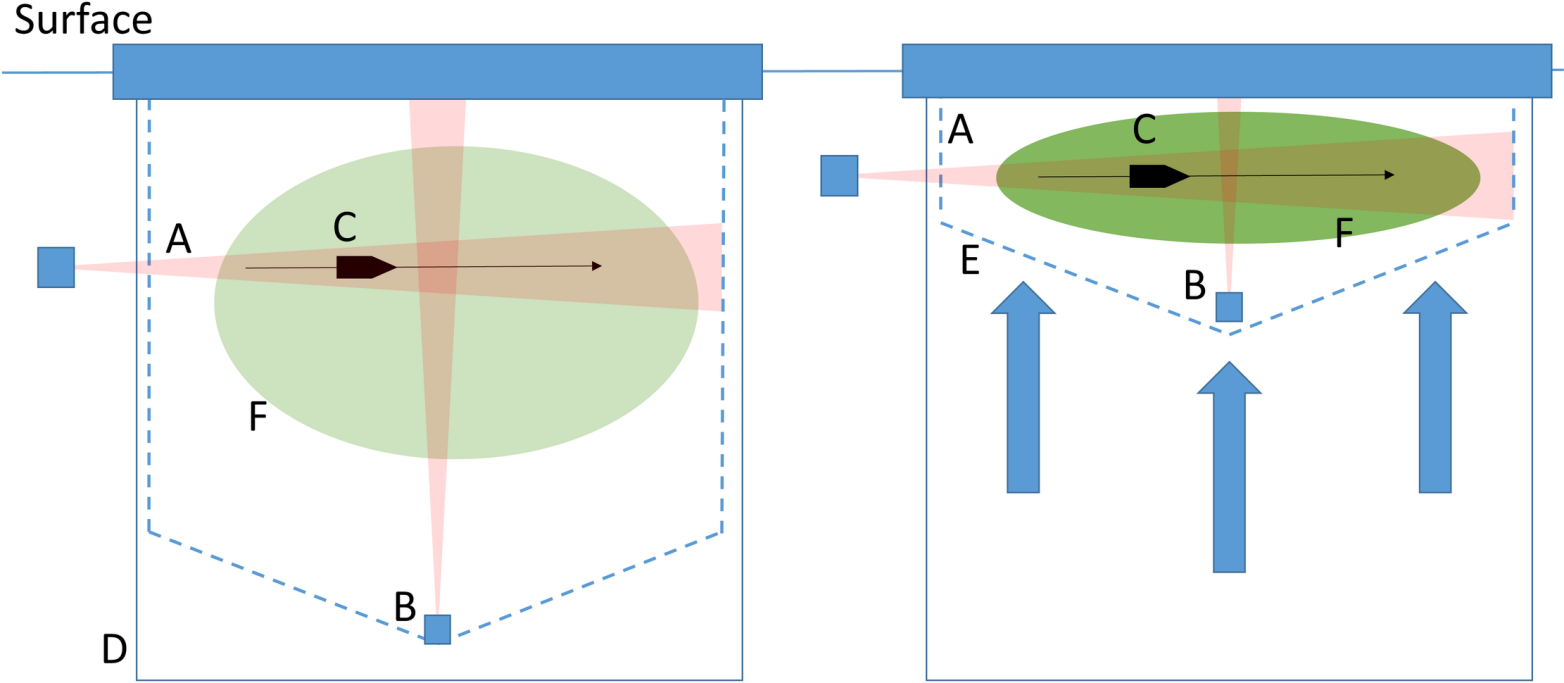
Vertical cross-section of the net pen and observation methodology. The 5m x 5m x 5m experimental pens were instrumented with a horizontally aligned imaging sonar (A) and an upward looking echo-sounder (B). A predator model (C) was pulled across the pen at a depth similar to the sonar to test the ability of the fish to react during the different treatments. To simulate hypoxia, a tarpaulin bag was wrapped around the pen (D; solid line) to prevent replenishment of the oxygen. The crowding was simulated by lifting the bottom of the net (E; broken line) effectively restricting the volume and decreasing the fish density (F).

The fish were transferred from the holding pen and divided into 12 experimental pens of dimensions 5 m x 5 m x 5 m. There were 4 pens available for experimentation, so the experiment was conducted in three replicate phases (Fig 1, Table 1).

**Table 1:** Experimental design. Overview of the experimental pens. There were 2 pens for hypoxic only and four for hypoxic and crowding; otherwise balanced. The fish density (ρ) in numbers per unit volume is calculated from the average backscattering coefficient (sv) (See methods).

Treatment	Date	ρ	N	Δ*r*
		m^-3^		m
Replicate Group 1				
crowded hypoxic	08/09/15	37	705	1,3
crowded hypoxic	09/09/15	13	503	1,6
crowded	10/09/15	25	669	1,4
control	11/09/15	NA^*^	NA^*^	NA^*^
Replicate Group 2				
hypoxic	27/09/16	27	1282	1,5
crowded	28/09/16	62	951	0,5
control	29/09/16	21	1838	3,1
crowded hypoxic	30/09/16	78	1185	0,4
Replicate Group 3				
control	24/10/16	26	1891	2,8
hypoxic	25/10/16	44	1230	1,9
crowded	26/10/16	63	1662	1,0
Crowded hypoxic	27/10/16	63	1795	1,0

* Missing echo-sounder data

A tunnel made of netting was set up between the pens, and fish transfer was motivated by adding feed in the experimental pen just after adding feed in the holding pen (to allow for mixing of the fish prior to the transfer). Ideally the number of fish in each experimental pen should be the same, but it is not feasible to count each individual fish during transfer and some differences are to be expected. The fish were allowed to acclimatise for 7 days in the experimental pens before starting each replicate, and the fish were only exposed to a treatment once during the experiments. It was clear that this acclimation period was adequate, because the fish quickly re-established the schooling and feeding behaviours that had been evident in the holding pen.

Throughout the experiment, each cage was monitored daily for compliance with the animal welfare permit. The general status of the fish was monitored, and any dead or severely injured fish were removed. Observed deaths were considered to be captivity related, if they were observed pre-treatment or in the control cages. Otherwise, any observed mortality was considered to be treatment related; unless it could be directly attributed to captivity. If a cadaver was observed, but could not be retrieved (i.e. was not in the mort sock), it was left in the cage but was counted as a mortality on that day. Later retrieval of the cadaver would be subtracted from the mortality count for that respective collection day, only if it could be positively identified.

### Experimental design

The treatments were “control”, “hypoxia”, “crowding”, and “crowding and hypoxia”. Crowding was simulated by first letting the fish move freely in the experimental pen followed by lifting the bottom and sides of the experimental pen restricting the volume available for the mackerel (Fig 1E). The hypoxia was simulated by isolating the pen with a tarpaulin bag (usually used for chemical bath treatments), thus restricting any flow of oxygenated water into the pen (Fig 1D). To ensure comparability between treatments, a tarpaulin bag was set around every pen; however, in non-hypoxic treatments (i.e. control and crowding) the bag remained open thus allowing an exchange of oxygenated water with the surrounding water-mass.

Each experimental pen was monitored several times during the experiment (denoted sampling times in the following). Sample time was measured in hours after the start of the treatment. The behavioural parameters were monitored once before (sample time = 0) and three times during exposure. For the 2015 experiment, post-exposure behaviour was monitored at day 1 and 6, to assess how the behavioural response recovered over time. In the 2016 experiments, post-exposure monitoring sample times was increased to 0.5, 2, and 4 hours (after last exposure) and consecutive days from day one to five days after exposure, to address concerns about poor time resolution. The dynamic response to the predator model was not measured at day two and three post treatment due to logistical challenges. At the end of the 5-day monitoring period, the fish were transferred into a second holding pen and held until the end of the experiment. The termination of the experiment was conducted in line with animal welfare regulations.

### Measuring the stressors

#### Oxygen concentration

Dissolved oxygen concentration was continuously measured using a RINKO III oxygen sensor (accuracy ±2% at 25°C) and extracted for each sample time. The concentration of dissolved oxygen in seawater depends on salinity, temperature, atmospheric pressure and depth [29]. Observed baseline oxygen concentrations, pre- and post-treatments ranged from 7.229 to 8.318 mg.l^-1^, while oxygen minimums during hypoxia treatments ranged between 2.811 and 3.345 mg.l^-1^. To aid comparability between treatments in this experiment, dissolved oxygen measurements are presented as percentages of equilibrated air-saturation in the results section.

#### Abundance, vertical fish distribution and fish density

**Fig 2:**
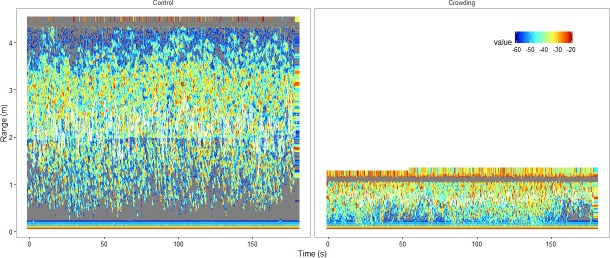
Example echogram. *Examples of echograms during control (left panel) and crowding (right panel) treatments*. *The depth is positive for increasing depth*. *The distance between the black lines shows the fish vertical distribution* Δ*r* (*m*), *excluding data closer than 50 cm from the transducer and echoes from the surface*. *The colour scale indicates echo strength*, *S*_*v*_ *(dB re 1 m*^*-1*^*)*, *which equals* 10 log_10_(*s*_*v*_).

Fish abundance (N), vertical distribution (Δ*r*) and density (ρ) inside the pen was measured using a Simrad EK60 echo sounder connected to a Simrad ES120-7C 120 kHz transducer (Kongsberg Maritime AS, Norway) (Table 1, Fig 2). The measures were calculated for each sample time. The transducer power was set to 105 W, ping repetition frequency 5 s^-1^, and pulse duration varied between 64 μs and 256 μs. The echo sounder was calibrated on both pulse lengths using a standard 22 mm tungsten carbide sphere [30] after the experiments.

The fish vertical range Δ*r* was defined as the distance from the lower to the upper part of the fish registration (c.f. Fig 2) and the density was calculated by $\rho =\bar{s_{v,i}}/\sigma_{bs}$, where $\bar{s_{v}}$ is the mean volume backscattering coefficient within the vertical range Δ*r*, and *σ*_*bs*_ is the backscattering cross-section of individual mackerel. The backscattering strength was derived from measured target strength *TS* = 10log_10_(*σ*_*bs*_) for 38 kHz [31], which is

$$TS[38\;kHz]=20log_{10}(L)-86.0\;dB$$

and the relative frequency response between 38 kHz and 120 kHz, which has been measured to be r = 1.5 [32], resulting in the target strength for 120 kHz being

$$TS[120\;kHz]=20log_{10}(L)-86.0+10log10(1.5)dB=20log_{10}(L)\text{-}80.4\;dB$$

where *L* is the average fish length. The estimated abundance was obtained using conventional echo integration, where the abundance *N* within the pen was approximated by

$$N=\frac{\bar{s_{v,i}}}{\bar{\sigma_{bs,i}}}\Delta r\cdot A,$$

where *A* is the surface area of the pen.

If the target strength is biased, the density and abundance will also be biased. For abundance, we also assume a uniform horizontal distribution, which is not likely the case. The numbers should therefore be taken as approximations of the abundances and densities. The relative measures, e.g. the relative increase/decrease in density during a trial, are robust since both the TS and the area cancel when calculating the ratios.

### Measuring the behavioural response

**Fig 3:**
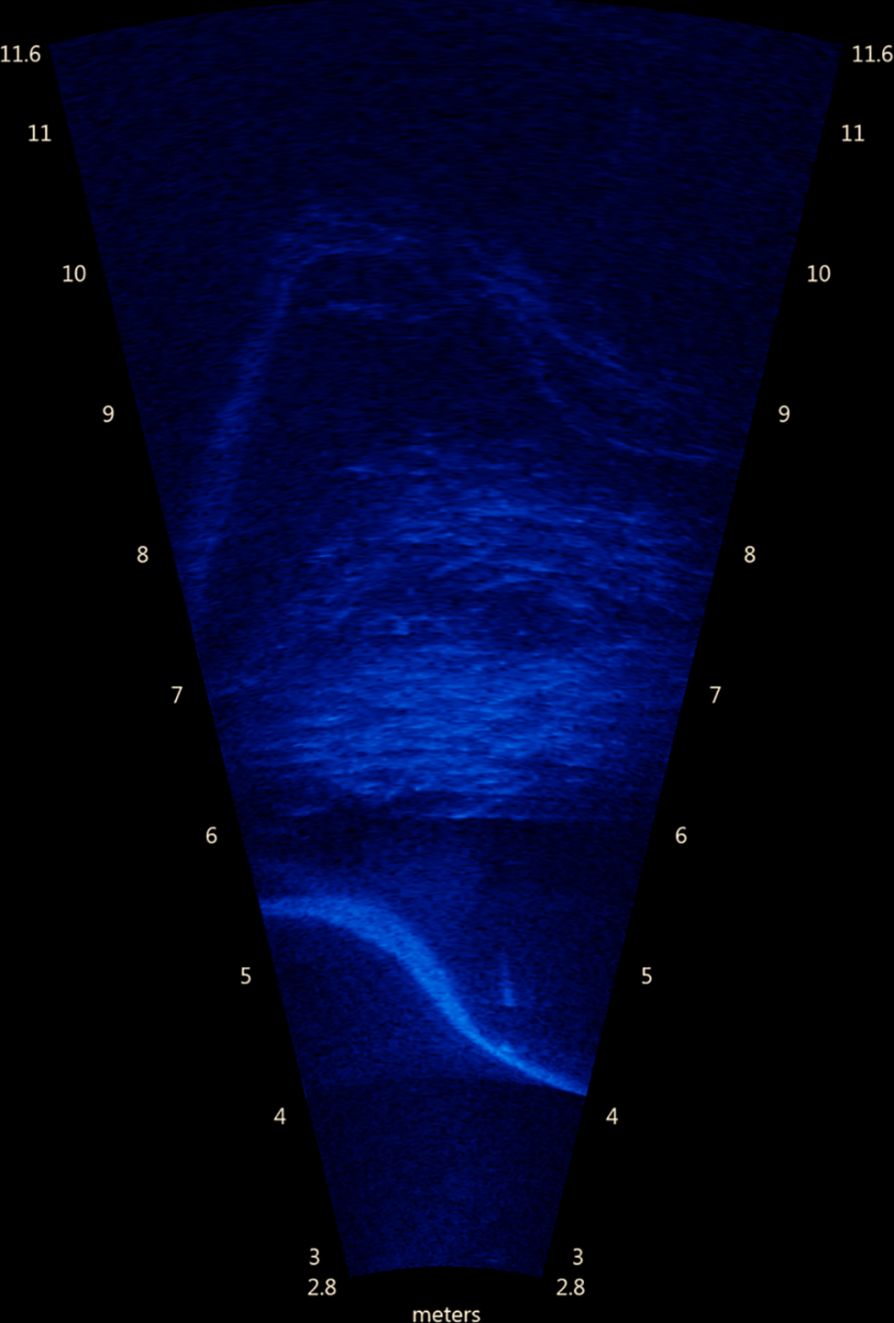
Image from the imaging sonar. A random image from the imaging sonar. The net pen walls are the slightly curved outline and the school is the circular pattern in the centre. Four video sequences (S1–S4 Videos) were randomly picked from the material showing responses scored as 0, 1, 2 and 3, respectively.

A horizontally aligned imaging sonar (ARIS 1800, Soundmetrics Corp., US, WA) was placed at approximately 1.8 m depth for the non-crowded case and approximately 1 m depth during crowding, aligning the beams horizontally across the pen (Fig 1). The sonar has 96 beams in a horizontal fan spanning 28°, resulting in a between beam spacing of 0.3° corresponding to a between beam resolution of 25 mm at 5 m range. The vertical opening angles are 14°. The sonar was operated at 1.8 MHz giving a range resolution of 3 mm and a frame rate of 15 frames per second, resulting in a high-resolution image sequences of the fish distribution across the pen (Fig 3). The imaging sonar is ideal for describing the swimming behaviour, since it covers the fish distribution across the pen.

#### Swimming speed

For each experimental pen and sampling time, a 30 s time window prior to predator model exposure (see below) was recorded, and an optical flow algorithm designed to track the schooling patterns of schooling fish [22,28] was used to estimate the average swimming velocity. The densities were too high for traditional tracking methods [33], and the optical flow algorithm estimates the fish flow velocities as opposed to tracks and velocities of individual fish. The average speed was derived from the velocities.

#### Dynamic schooling response

To test the indirect implications of the treatment on the schooling function in mackerel, we tested the dynamic schooling response to an approaching predator model. A predator shaped model was pulled across the school approximately at the depth of the imaging sonar [26] (Fig 1C), and four consecutive model exposures were conducted for each trial and sample time. The reason for using four consecutive treatments for each sample time was to establish whether there was a habituation effect or not. We exported the image frames from the imaging sonar in a time window 20 s prior to and 10 s after the predator model treatment. This resulted in 4 sonar video sequences for each trial and sampling time. The sequences were manually scored by an evaluation panel consisting of three persons. The sequences were presented in random order and the reaction were categorized from no reaction (score equal to zero) to a strong reaction (score equal to three). A strong reaction was defined as a collective response affecting a substantial proportion of the fish within the field of view of the sonar. Examples of typical video sequences (randomly chosen within each category) for the different categories are given in supplementary material (S1–S4 Videos). The average score over the panel members was used as the response variable for a given video sequence.

### Data analysis

The response variables (score and swimming speed) was modelled using a generalized linear model (GAM) and fitting a smoothing spline as a function of sample time by treatment (control, hypoxic, crowding, or crowding and hypoxic). GAMs allow to 1) model both parametric effects and non-parametric effects through a smoothing function and 2) provide a framework to account for nonlinear and non-monotonic relationships between response variables and a set of explanatory variables [34,35]. We used the *gam* function from the *mgcv* package in the R environment to fit the data to the model, i.e. score ~ s(log(time+1),by = type) and speed ~ s(log(time+1),by = type), where type is the treatment and time is the sample time in hours. An analysis of variance was used to test which of the fitted model parameters by type were significant.

The GAM curves can be reviewed to assess when the response occurs in the sample time series. However, the GAM does not explicitly test when in the time series any response occurs. To test differences between the treatment types *during exposure*, i.e. during the control, when the fish is crowded, or when the oxygen is reduced, we used a Kruskal-Wallis test followed by a *post hoc* test between all treatment types (Nemenyi test). This was used to confirm the visual inspections from the GAM model.

## Results

### The treatments

**Fig 4:**
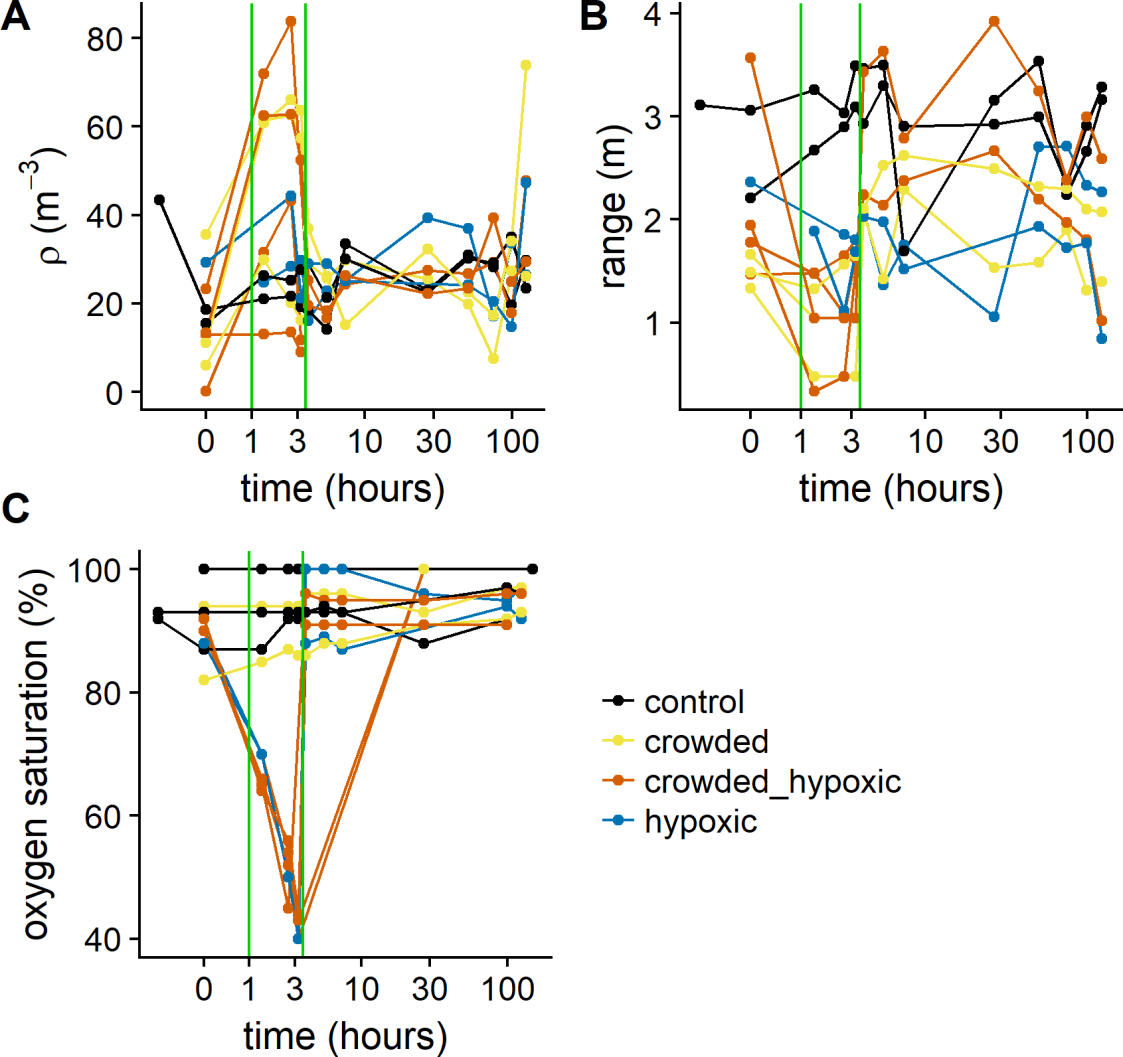
The exposures as a function of sample time. (A) the average fish density calculated from the upward looking echo-sounder, (B) the range defined as the upper minus the lower boundary of the fish distribution within the pen detected by the echo-sounder, and (C) the dissolved oxygen saturation (%) for each treatment. Each line represents one treatment (one line in Table 1), and the colour indicates the treatment type. The time is given in hours after start of treatment. The green vertical lines represent the treatment time period.

The estimated abundance in each pen is given in Table 1. The number of fish in each pen was generally lower during the first year (Replicate Group 1), compared to the second year (Replicate Groups 2 & 3), although there was variability between the pens. During year 1, the abundance estimated by the acoustics varied between 500 and 700 fish and for the second year the abundance varied between 950 and 1900 fish. The change in density (Fig 4A) and vertical range (Fig 4B) during the treatments show an average 2.4 times increase in density during the crowding and crowding and hypoxia treatments compared to the control and hypoxia only.

The hypoxia treatment setup worked well, and oxygen concentrations were brought down to ~40% saturation (Fig 4C). The difference in number of fish in the pens affected the rate at which the depletion occurred, and the time from >90% to 40% took typically 3 hours; but was as low as 1.5 hours due to high fish abundance in the pen (pen D; Replicate Group 3). To align the oxygen levels for the analysis during exposure, the actual sample times was replaced by the average sample times, i.e. 1.4, 2.6 and 3.2 hours, instead of the actual sample times.

### The behavioural responses

#### Swimming speed

The swimming speeds at each sampling point (prior to the predator model treatments) did not show any clear differences or patterns between the hypoxic and control treatments (S1 Fig). During crowding, the fish density got to a point where the optical flow failed, and comparisons between the crowded and non-crowded cases were therefore not possible. For the hypoxia cases, there is no clear signal (Approximate significance of GAM smooth terms, p = 0.7).

#### Dynamic schooling response

For the dynamic schooling responses, we did 4 consecutive predator model exposures for each sampling point, separated by 2 minutes. We did this to test if there was any habituation to repeated exposures. We fitted a linear curve to the score of the 4 subsequent reactions, and the results show that the average score-reduction per treatment was approximately -0.05 score-points and that the number is significantly different from zero (p = 0.008, t-test). This means that the reaction drops on average 0.2 points during the four consecutive treatments. However, the reduction is minor compared to the range of the scores (0–3), and we chose to use the average over the four consecutive treatments as the score for each sampling point in the following analysis.

**Fig 5:**
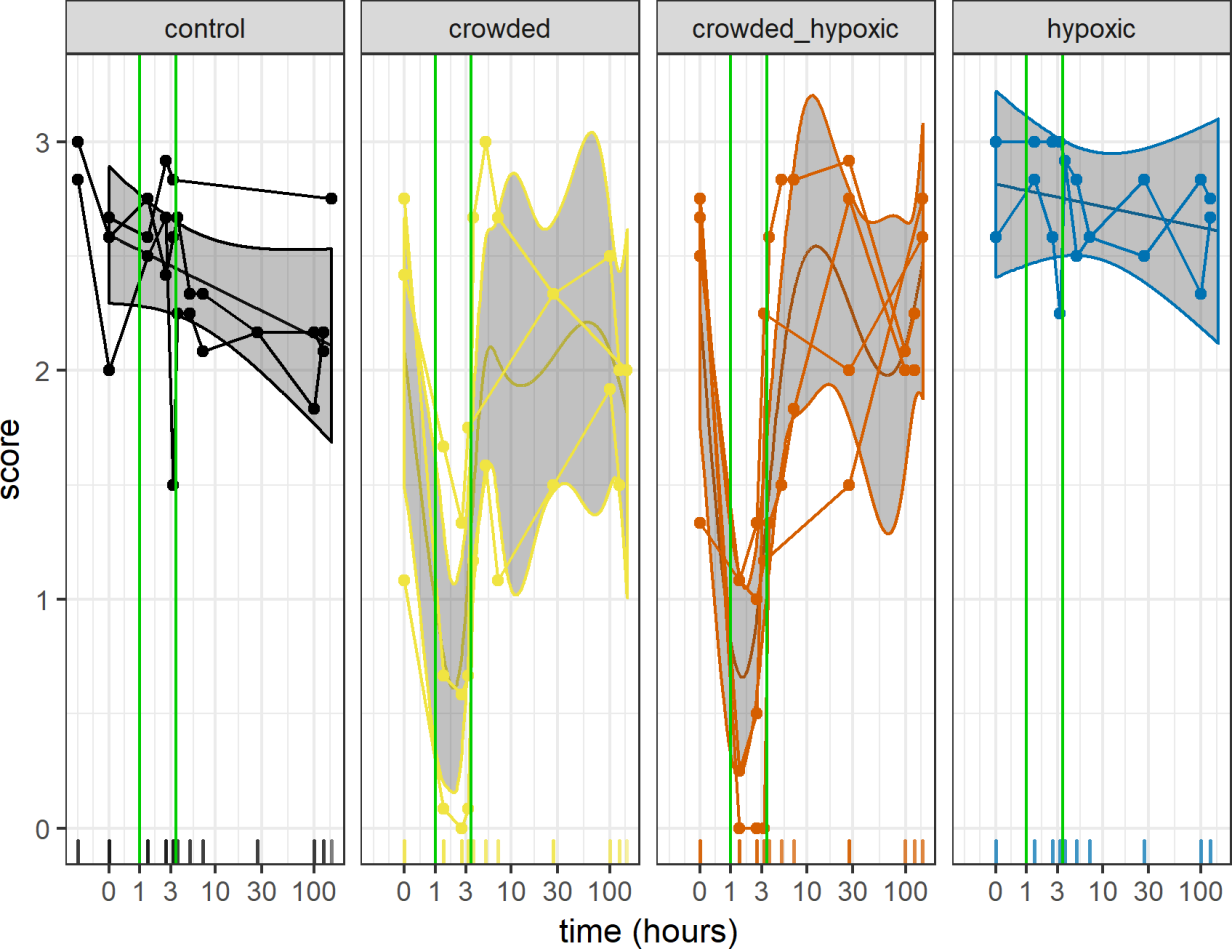
The dynamic reaction score to the predator model as a function of sample time. The curves represent the GAM model fitted to the data with 95% CI intervals. The dots connected with curves represents one treatment (one line in Table 1), and the colour indicates the treatment type. The time is given in hours from the start of the treatment. The green vertical lines represent the treatment time interval, c.f. Fig 4.

**Fig 6:**
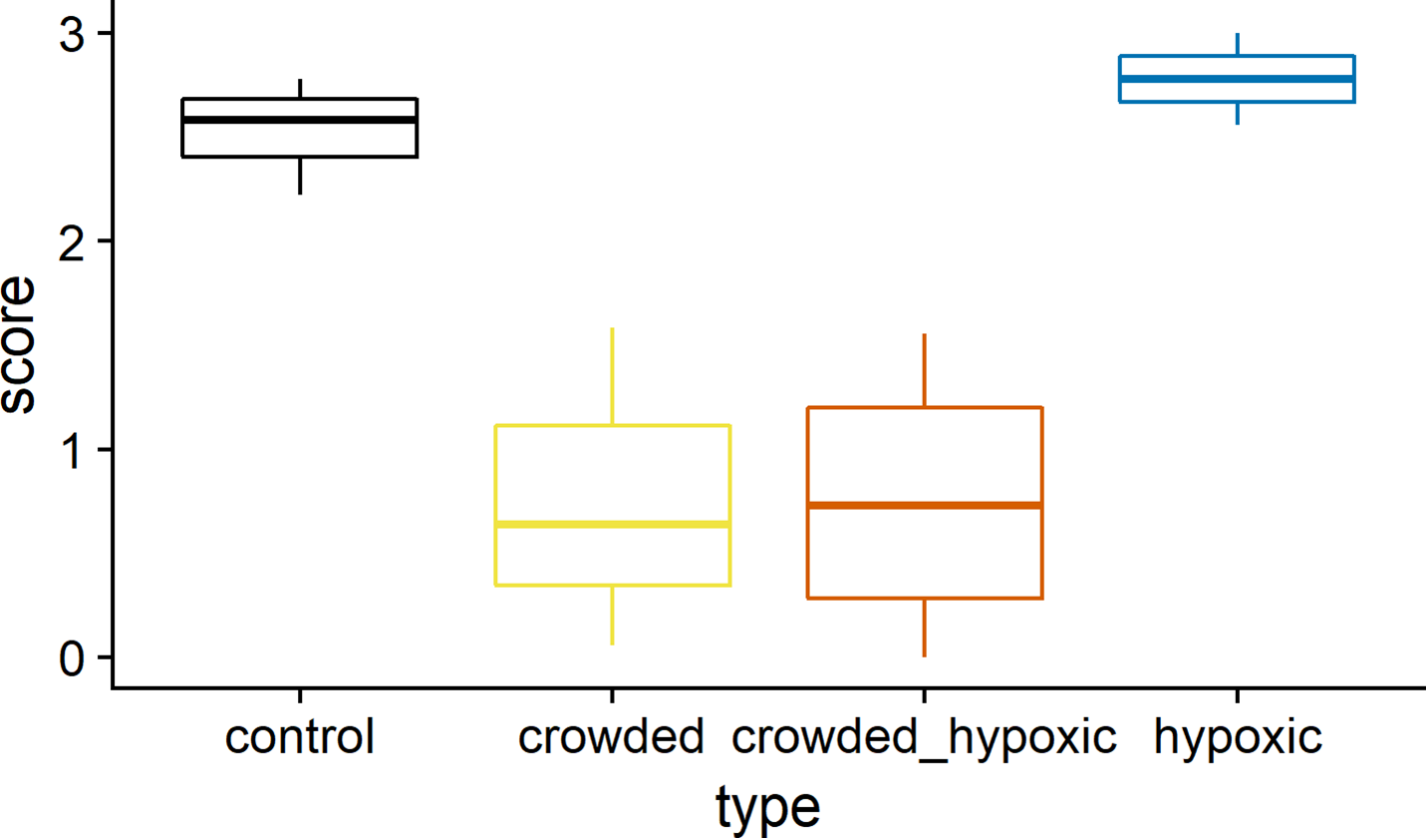
The dynamic reaction score during exposure. The lower and upper hinges correspond to the first and third quartiles and the line is the 1.5 times the interquartile range.

The dynamic schooling response to the predator model, as a function of treatment, shows that there is a strong reduction in dynamic schooling response during crowding, whereas the response was not affected in the control and hypoxia treatments (Fig 5). The approximate significance of the GAM smooth terms show a significant effect of the crowded (p<0.001) and crowded-hypoxic treatment types (p>0.001). This test does not establish when in the time series the change occurs, but visual inspection of the smooth term confirms that it coincides with the treatment (Fig 5). To formally test this, we used the average score for each experimental pen over the treatment as the response, and we found significant differences between the treatments (Kruskal-Wallis chi-squared = 8.1859, df = 3, p = 0.04). A *post hoc* test between all treatments shows that the control vs hypoxia treatments (Nemenyi test, p = 0.99) and crowded vs crowded and hypoxic treatments (Nemenyi test, p = 1.00) are very similar (Fig 6). When merging these similar groups, the difference between the crowded and non-crowded cases are significantly different (Kruskal-Wallis chi-squared = 8.0769, df = 1, p-value = 0.004), which confirms the visual impression from the GAM model.

Some of the crowded groups’ responses immediately returned to pre-crowded levels while other groups appeared to have a delayed return. This seems to be the case when the response to the crowding was stronger (score < 0.2). Again, the variability is large, and the score regressed against time after the treatment (time>3.2 hours) showed no significant increase in score after the treatment (linear regression, p = 0.67).

### Confirming sub-lethal exposures

Fifty-one mackerel (out of ~14 000 in total) died during the experiment, particularly in Replicate Group 3 (S1 Table), confirming that the exposures were close to sub-lethal. These deaths could be attributed to both captivity effects and the treatments, and were typically associated with skin lesions. Captivity related mortality (i.e. pre-treatment and in control cages) appears to have increased with increasing sample size in the cages and, in the controls, was typically observed early in the monitoring period. There were two conspicuous treatment related mortality events, both in Replicate Group 3: Cage B (Crowding) had a mortality proportion of 0.0018 (Confidence interval (CI): 0.0006, 0.0053) and Cage D (Crowding & hypoxia) 0.011 (CI: 0.0072, 0.017). Both events were associated with the densest crowding treatments observed in the experiment (c.f. Table 1). However, neither of these events were significantly distinguishable from captivity related mortality; either pre-treatment or from their respective control.

## Discussion

This experiment has demonstrated that changes in behaviour (schooling function) were observable at sub-lethal levels of a capture related stressor: crowding. However, no significant changes in behaviour were observed at sub-lethal levels of hypoxia. The net pen setup allowed a high number of individuals with reasonable control of the stressors, and so offered a compromise in realism between the fishing operation and the laboratory setting.

The main challenge was controlling the number of fish in the experimental pens, as it was not possible to accurately count fish during transfer. This led to differences in population size with the different pens. For the hypoxia treatment, the variation in biomass meant the rate at which oxygen was consumed varied. Some pens took almost three hours to deplete oxygen concentrations to the pre-determined endpoint of 40% saturation. Thus, the oxygen reduction rates are likely lower than in commercial operations. We have not observed oxygen concentrations as low as 40% saturation, even in catches of several hundred tonnes (pers. Comm. Michael Breen). Consequently, the levels we used were realistic. For the crowding treatment, the variation in population sizes caused a difference in crowding density between pens. However, there was a clear increase in the densities during the crowding treatment and a strong and significant signal in the behavioural responses, and, consequently, the result that crowding inhibits the schooling response is robust to the varying number of fish in the experimental pens.

The failure of the PIV algorithm on the high densities prohibited us from investigating the effect on the crowding treatment, but assessing differences in swimming speed between the control and the hypoxia treatment was possible. Changes in swimming speed has previously been reported as a dynamic response in schooling fish exposed to stressors [24,28], and increased swimming speeds have been demonstrated to be an efficient strategy for schooling fish to avoid unfavourable areas and to climb gradients [19]. There seemed to be an increasing trend with decreased oxygen, which would corroborate these earlier findings [36,37], but the signal is not significant. This could possibly be addressed with a higher sample size, but the variability between the pens after treatment is high. Ground truthing of the PIV estimates using cameras would also be useful.

The mackerel in this experiment were held in captivity for extended periods; almost 12 months for Replicate Group 1. Therefore, it is possible that acclimation to captive conditions in the pens may have desensitised these fish to stimuli presented to them during the crowding treatment [38]. Whereas, wild fish would likely respond sooner, and arguably more strongly, to the novel stimuli presented by being crowded inside a net. However, this does not undermine our hypothesis that behaviour responses to sub-lethal stressors could be used as early warning indicators, to avoid slipping related mortality. Indeed, a more rapid response from wild fish would suggest that fatal levels of crowding stress would more likely be avoided.

The repetitive exposure of stimuli during short intervals of time may cause focal experimental individuals to become habituated to the presented stimuli leading to reduced reactions [40]. Our results indicate a negligible reduction in mackerels' responses to the consecutive predator model treatments. Previous studies have demonstrated that schooling fish (wild-caught Atlantic herring, *Clupea harengus*) repetitively exposed to a similar predator model did not show reduced collective responses over time, and that they were able to regain a "normal" pre-exposure collective swimming dynamics and school internal organisation only few minutes (< 3 min) after exposure [26,41,42]. In addition, the greater time intervals of exposure to the predator model during the post-treatment phase may have contributed to prevent the risk of an habituation effect, as the timing of exposure was unpredictable for the fish (as suggested by earlier work [43]). It is also worth noting that, due to the number of individuals present in each experimental net pen and their dynamic swimming pattern, it is likely that there was a substantial mixing of individuals ensuring that not the same few individuals directly reacted to the predator model alone each time [28], as opposed to the subsequent waves of behavioural responses. It is, consequently, not likely that the (lack of) reactions observed after treatment have been caused by repeated exposures to the same individuals.

During the treatments, we followed the protocol and evaluated whether the crowding densities were within safe limits during the experiment. We assessed this based on the acoustic and optical images combined with guidance from the experienced keepers. Subsequent post-processing of the data revealed some cases where the predefined threshold was exceeded. This may be caused by an underestimate of the target strength and subsequent overestimate of the abundance. Although the treatment mortalities were low, an improved method to monitor the treatment densities would be useful.

The mortality of mackerel observed in this experiment was generally small (<1.5% of the observed cage population) and could be attributed primarily to captivity, but also to some treatments (crowding and crowding & hypoxia). In addition, there were 5 instances were dead fish were observed, but not subsequently identified, and so may have been double counted. As such, these mortality estimates should be considered as the maximum likely observed mortality in these experiments. The observed deaths were typically delayed, starting 2–3 days after treatment, and associated with severe skin lesions, which agrees with our pilot study observations on the effects of skin injury [44]. The captivity related mortality also appeared early in the monitoring period, at least in the controls, and this suggests that these deaths were most likely related to increased risk of abrasive injury (particularly with larger population sizes) during the transfer into the cages. In summary, these experiments have observed crowding treatments up to a potentially fatal threshold for a small proportion of the population (<1%). However, no specifically hypoxia related fatalities were observed. Therefore, behavioural changes observed as these thresholds were approached, could be used as an early, sub-lethal, indicator of stressful conditions in purse seine catches.

The crowding treatment induced a strong change in schooling behaviour, and we have not established the mechanism that inhibits the response. One explanation could be a reduced opportunity to respond since the space is restricted. Another explanation is that the decreased inter-individual spacing is causing the lack of the response. The amplification of behavioural perturbations is related to the sensory information the individual fish detect [39], and a high density may restrict the visual field to such an extent that only a few individuals detect and respond to the threat. This can inhibit the amplification of behavioural waves, which have been observed in schools where the schooling behaviour is intact [27].

## Conclusions

The objective of this study was to find sub-lethal behavioural indicators of potentially fatal capture related stressors (crowding and hypoxia) that could be used to avoid slipping induced mortality in purse seine fishing. The strongest sub-lethal behavioural signal was a reduced response to a predator model at high crowding densities. Unfortunately, this is not a particularly practical measure to obtain during commercial fishing operations. However, it did demonstrate a measurable and reproducible change in behaviour at sub-lethal levels of a normally fatal stressor. The challenge now is to be able to practically monitor the behaviour and devise some technology to reliably measure this change. Another useful result was the verification, at a large scale, of sub-lethal thresholds for crowding and hypoxia used in the experiment; where schools of mackerel survived crowding up to 100 m^-3^ (~88 kg m^-3^) and hypoxia saturations down to 40% without a significant increase in mortality.

## Supporting information

S1 Table The fish mortalities during the experiment. (PDF)

S1 Fig The swimming speed as a function of time. (TIF)

S1 Video Randomly chosen video sequence among responses scored as 0. (MP4)

S2 Video Randomly chosen video sequence among responses scored as 1. (MP4)

S3 Video Randomly chosen video sequence among responses scored as 2. (MP4)

S4 Video Randomly chosen video sequence among responses scored as 3. (MP4)

S1 Data set Contains data to re-generate the tests and the figures in the paper. (CSV)

S1 R script R script to read the supporting data set for regenerating the tests and figures in the paper. (R)

## References

[1] Watson R, Revenga C, Kura Y. Fishing gear associated with global marine catches: I. Database development. Fisheries Research. 2006;79: 97–102. doi: 10.1016/j.fishres.2006.01.010

[2] Ben-Yami M. Purse seining manual Oxford; Cambridge, MA, USA: Published by arrangement with the Food and Agriculture Organization of the United Nations (FAO) by Fishing News Books; 1994.

[3] Suuronen P, Chopin F, Glass C, Løkkeborg S, Matsushita Y, Queirolo D, et al Low impact and fuel efficient fishing—Looking beyond the horizon. Fisheries Research. 2012;119–120: 135–146. doi: 10.1016/j.fishres.2011.12.009

[4] Tenningen M, Macaulay GJ, Rieucau G, Peña H, Korneliussen RJ. Behaviours of Atlantic herring and mackerel in a purse-seine net, observed using multibeam sonar. ICES J Mar Sci. 2017;74: 359–368. doi: 10.1093/icesjms/fsw159

[5] Stratoudakis Y, Marçalo A. Sardine slipping during purse-seining off northern Portugal. ICES J Mar Sci. 2002;59: 1256–1262. doi: 10.1006/jmsc.2002.1314

[6] Gezelius SS. Monitoring fishing mortality: Compliance in Norwegian offshore fisheries. Marine Policy. 2006;30: 462–469. doi: 10.1016/j.marpol.2005.06.004

[7] Huse I, Vold A. Mortality of mackerel (Scomber scombrus L.) after pursing and slipping from a purse seine. Fisheries Research. 2010;106: 54–59. doi: 10.1016/j.fishres.2010.07.001

[8] Tenningen M, Vold A, Olsen RE. The response of herring to high crowding densities in purse-seines: survival and stress reaction. ICES J Mar Sci. 2012;69: 1523–1531. doi: 10.1093/icesjms/fss114

[9] Mesnil B. When discards survive: Accounting for survival of discards in fisheries assessments. Aquat Living Resour. 1996;9: 209–215. doi: 10.1051/alr:1996024

[10] Breen M, Cook R. Inclusion of discard and escape mortality estimates in stock assessment models and its likely impact on fisheries management. ICES CM. 2002;27: 15.

[11] Viana M, McNally L, Graham N, Reid DG, Jackson AL. Ignoring discards biases the assessment of fisheries’ ecological fingerprint. Biology Letters. 2013;9: 20130812 doi: 10.1098/rsbl.2013.0812 24307530 10.1098/rsbl.2013.0812 PMC3871369

[12] Anon. Act of 6 June 2008 no. 37 relating to the management of wild living marine resources (“The marine resources act”). Norwegian Ministry of Trade, Industry and Fisheries; 2008.

[13] European Union. Regulation (EU) No 1380/2013 of the European Parliament and of the Council of 11 December 2013 on the Common Fisheries Policy, amending Council Regulations (EC) No1954/2003 and (EC) No1224/2009 and repealing Council Regulations (EC) No 2371/2002 and (EC) No 639/2004 and Council Decision 2004/585/ EC.,. L354: 22–61. Official Journal of the European Union; 2013.

[14] Misund OA. Avoidance behaviour of herring (Clupea harengus) and mackerel (Scomber scombrus) in purse seine capture situations. Fisheries Research. 1993;16: 179–194. doi: 10.1016/0165-7836(93)90051-8

[15] Tenningen M, Peña H, Macaulay GJ. Estimates of net volume available for fish shoals during commercial mackerel (Scomber scombrus) purse seining. Fisheries Research. 2015;161: 244–251. doi: 10.1016/j.fishres.2014.08.003

[16] Mitchell RW, Blight SJ, Gaughan DJ, Wright IW. Does the mortality of released Sardinops sagax increase if rolled over the headline of a purse seine net? Fisheries Research. 2002;57: 279–285. doi: 10.1016/S0165-7836(01)00354-X

[17] Lockwood SJ, Pawson MG, Eaton DR. The effects of crowding on mackerel (Scomber scombrus L.)—Physical condition and mortality. Fisheries Research. 1983;2: 129–147. doi: 10.1016/0165-7836(83)90114-5

[18] Marçalo A, Marques TA, Araújo J, Pousão-Ferreira P, Erzini K, Stratoudakis Y. Fishing simulation experiments for predicting the effects of purse-seine capture on sardine (Sardina pilchardus). ICES J Mar Sci. 2010;67: 334–344. doi: 10.1093/icesjms/fsp244

[19] Berdahl A, Torney CJ, Ioannou CC, Faria JJ, Couzin ID. Emergent Sensing of Complex Environments by Mobile Animal Groups. Science. 2013;339: 574–576. doi: 10.1126/science.1225883 23372013 10.1126/science.1225883

[20] Couzin ID, Krause J, Franks NR, Levin SA. Effective leadership and decision making in animal groups on the move. Nature. 2005;433: 513–516. doi: 10.1038/nature03236 15690039 10.1038/nature03236

[21] Hemelrijk C, Reid D, Hildenbrandt H, Padding J. The increased efficiency of fish swimming in a school. Fish Fish. 2015;16: 511–521. doi: 10.1111/faf.12072

[22] Handegard NO, Boswell KM, Ioannou CC, Leblanc SP, Tjøstheim DB, Couzin ID. The Dynamics of Coordinated Group Hunting and Collective Information Transfer among Schooling Prey. Current Biology. 2012;22: 1213–1217. doi: 10.1016/j.cub.2012.04.050 22683262 10.1016/j.cub.2012.04.050

[23] Treherne JE, Foster WA. Group transmission of predator avoidance behaviour in a marine insect: The trafalgar effect. Animal Behaviour. 1981;29: 911–917. doi: 10.1016/S0003-3472(81)80028-0

[24] Marçalo A, Araújo J, Pousão-Ferreira P, Pierce GJ, Stratoudakis Y, Erzini K. Behavioural responses of sardines Sardina pilchardus to simulated purse-seine capture and slipping. J Fish Biol. 2013;83: 480–500. doi: 10.1111/jfb.12184 23991869 10.1111/jfb.12184

[25] Domenici P, Lefrancois C, Shingles A. Hypoxia and the antipredator behaviours of fishes. Phil Trans R Soc Lond B. 2007;362: 2105–2121. doi: 10.1098/rstb.2007.2103 17472921 10.1098/rstb.2007.2103 PMC2442856

[26] Rieucau G, Boswell KM, De Robertis A, Macaulay GJ, Handegard NO. Experimental Evidence of Threat-Sensitive Collective Avoidance Responses in a Large Wild-Caught Herring School. PLoS ONE. 2014;9: e86726 doi: 10.1371/journal.pone.0086726 24489778 10.1371/journal.pone.0086726 PMC3906054

[27] Rieucau G, Sivle LD, Handegard NO. Herring perform stronger collective evasive reactions when previously exposed to killer whales calls. Behavioral Ecology. 2016;27: 538–544. doi: 10.1093/beheco/arv186

[28] Rieucau G, Holmin AJ, Castillo JC, Couzin ID, Handegard NO. School level structural and dynamic adjustments to risk promote information transfer and collective evasion in herring. Animal Behaviour. 2016;117: 69–78. doi: 10.1016/j.anbehav.2016.05.002

[29] Benson BB, Krause D. The concentration and isotopic fractionation of oxygen dissolved in freshwater and seawater in equilibrium with the atmosphere1. Limnology and Oceanography. 1984;29: 620–632. doi: 10.4319/lo.1984.29.3.0620

[30] Foote KG, Knudsen HP, Vestnes G, MacLennan DN, Simmonds EJ. Calibration of acoustic instruments for fish density estimation: A practical guide. 1987 p. 69. Report No.: ICES CRR No. 144.

[31] Fernandes P, Korneliussen R, Lebourges-Dhaussy A, Massé J, Iglesias M, Diner N, et al The SIMFAMI project: Species Identification Methods from Acoustic Multifrequency Information. Final report to the EC. 486 pp. 2006 p. 486 pp. Report No.: No. Q5RS-2001-02054.

[32] Korneliussen RJ. The acoustic identification of Atlantic mackerel. ICES J Mar Sci. 2010;67: 1749–1758. doi: 10.1093/icesjms/fsq052

[33] Handegard NO, Williams K. Automated tracking of fish in trawls using the DIDSON (dual frequency identification sonar). ICES Journal of Marine Science. 2008;65: 636–644. doi: 10.1093/icesjms/fsn029

[34] Wood SN. Generalized Additive Models: An Introduction with R In: CRC Press [Internet]. 27 2 2006 [cited 11 Oct 2017]. Available: https://www.crcpress.com/Generalized-Additive-Models-An-Introduction-with-R/Wood/p/book/9781584884743

[35] Guisan A, Edwards TC, Hastie T. Generalized linear and generalized additive models in studies of species distributions: setting the scene. Ecological Modelling. 2002;157: 89–100. doi: 10.1016/S0304-3800(02)00204-1

[36] Morgan RL. Using behaviour of herring (Clupea harengus L.) to assess post-crowding stress in purse-seine fisheries. 2014; Available: https://bora.uib.no/handle/1956/8338

[37] Howarth K. Assessing mackerel behaviour following crowding-induced stress in purse seine fisheries. 2016; Available: https://bora.uib.no/handle/1956/12589

[38] Portz DE, Woodley CM, Cech JJ. Stress-associated impacts of short-term holding on fishes. Rev Fish Biol Fisheries. 2006;16: 125–170. doi: 10.1007/s11160-006-9012-z

[39] Strandburg-Peshkin A, Twomey CR, Bode NWF, Kao AB, Katz Y, Ioannou CC, et al Visual sensory networks and effective information transfer in animal groups. Current Biology. 2013;23: R709–R711. doi: 10.1016/j.cub.2013.07.059 24028946 10.1016/j.cub.2013.07.059 PMC4780851

[40] Eaton RC, Bombardieri RA, Meyer DL. The Mauthner-initiated startle response in teleost fish. J Exp Biol. 1977;66: 65–81. 870603 10.1242/jeb.66.1.65

[41] Handegard NO, Robertis AD, Rieucau G, Boswell K, Macaulay GJ. The reaction of a captive herring school to playbacks of a noise-reduced and a conventional research vessel. Can J Fish Aquat Sci. 2014;72: 491–499. doi: 10.1139/cjfas-2014-0257

[42] Rieucau G, De Robertis A, Boswell KM, Handegard NO. School density affects the strength of collective avoidance responses in wild-caught Atlantic herring Clupea harengus: a simulated predator encounter experiment. J Fish Biol. 2014;85: 1650–1664. doi: 10.1111/jfb.12520 25243659 10.1111/jfb.12520

[43] Schleidt WM, Shalter MD, Carawan TC. The Effect of Spatial Context on Habituation to a Predator Model. Zeitschrift für Tierpsychologie. 1983;61: 67–70. doi: 10.1111/j.1439-0310.1983.tb01326.x

[44] Kitsios E. The nature and degree of skin damage in mackerel (Scomber scombrus) following mechanical stress: Can skin damage lead to mortality following crowding in a purse seine? Master of Science Thesis, Bergen University. pp64. University of Bergen. 2016.

