# Supplementary material for: Effects on schooling function in mackerel of sub-lethal capture related stressors: Crowding and hypoxia
Source: PLoS One. 2017 Dec 28;12(12):e0190259. doi: 10.1371/journal.pone.0190259 (PMC5746257; doi:10.1371/journal.pone.0190259)
Supplement: S1 Table — (PDF) [file pone.0190259.s001.pdf]

Cage	Treatment	Treatment Start Date	Category	Monitoring (Days relative to Treatment)													Total dead	Sample size	Proportion dead	Lower 95% CI	Upper 95% CI
				-4	-3	-2	-1	0	1	2	3	4	5	6	7	8					
**Replicate Group 1**																					
5A	Hypoxia & Crowding	09.09.2015	Treatment														0	503	0.0000	0.0000	0.0076
			Captivity														0	503	0.0000	0.0000	0.0076
5B	Crowding	10.09.2016	Treatment														0	669	0.0000	0.0000	0.0057
			Captivity														0	669	0.0000	0.0000	0.0057
5C	Hypoxia & Crowding	08.09.2015	Treatment														0	705	0.0000	0.0000	0.0054
			Captivity													3*	3	705	0.0043	0.0014	0.0124
5D	Control	11.09.2015	Treatment														-	-	-	-	-
			Captivity														0	NA	0.0000	0.0000	0.0076
**Replicate Group 2**																					
12A	Hypoxia	27.09.2016	Treatment														0	1282	0.0000	0.0000	0.0030
			Captivity					(2*)			1$		1$				2	1282	0.0016	0.0004	0.0057
12B	Crowding	28.09.2016	Treatment														0	951	0.0000	0.0000	0.0040
			Captivity														0	951	0.0000	0.0000	0.0040
12C	Hypoxia & Crowding	30.09.2016	Treatment														0	1185	0.0000	0.0000	0.0032
			Captivity		1												1	1185	0.0008	0.0001	0.0048
12D	Control	29.09.2016	Treatment														-	-	-	-	-
			Captivity		1			1$	2$				1$				5	1838	0.0027	0.0012	0.0064
**Replicate Group 3**																					
12A	Hypoxia	25.10.2016	Treatment														0	1230	0.0000	0.0000	0.0031
			Captivity														0	1230	0.0000	0.0000	0.0031
12B	Crowding	26.10.2016	Treatment								1		2#				3	1662	0.0018	0.0006	0.0053
			Captivity			1$		2									3	1662	0.0018	0.0006	0.0053
12C	Control	24.10.2016	Treatment														-	-	-	-	-
			Captivity					3	1#			1		1#		1	7	1891	0.0037	0.0018	0.0076
12D	Hypoxia & Crowding	27.10.2016	Treatment							4(2#)	3	7	4				20	1795	0.0111	0.0072	0.0171
			Captivity		2	2	1#	2									7	1795	0.0039	0.0019	0.0080

NA Echosounder estimate unavailable. Assumed to be ~500 for 95% CI estimates.
* Fish caught in netting fold at termination of experiment
\# Observed but not retreived
$ Badly decomposed
Observation period in captivity
No Observations
