# Supplementary figures and images for: Effects on schooling function in mackerel of sub-lethal capture related stressors: Crowding and hypoxia
Source: PLoS One. 2017 Dec 28;12(12):e0190259. doi: 10.1371/journal.pone.0190259 (PMC5746257; doi:10.1371/journal.pone.0190259)

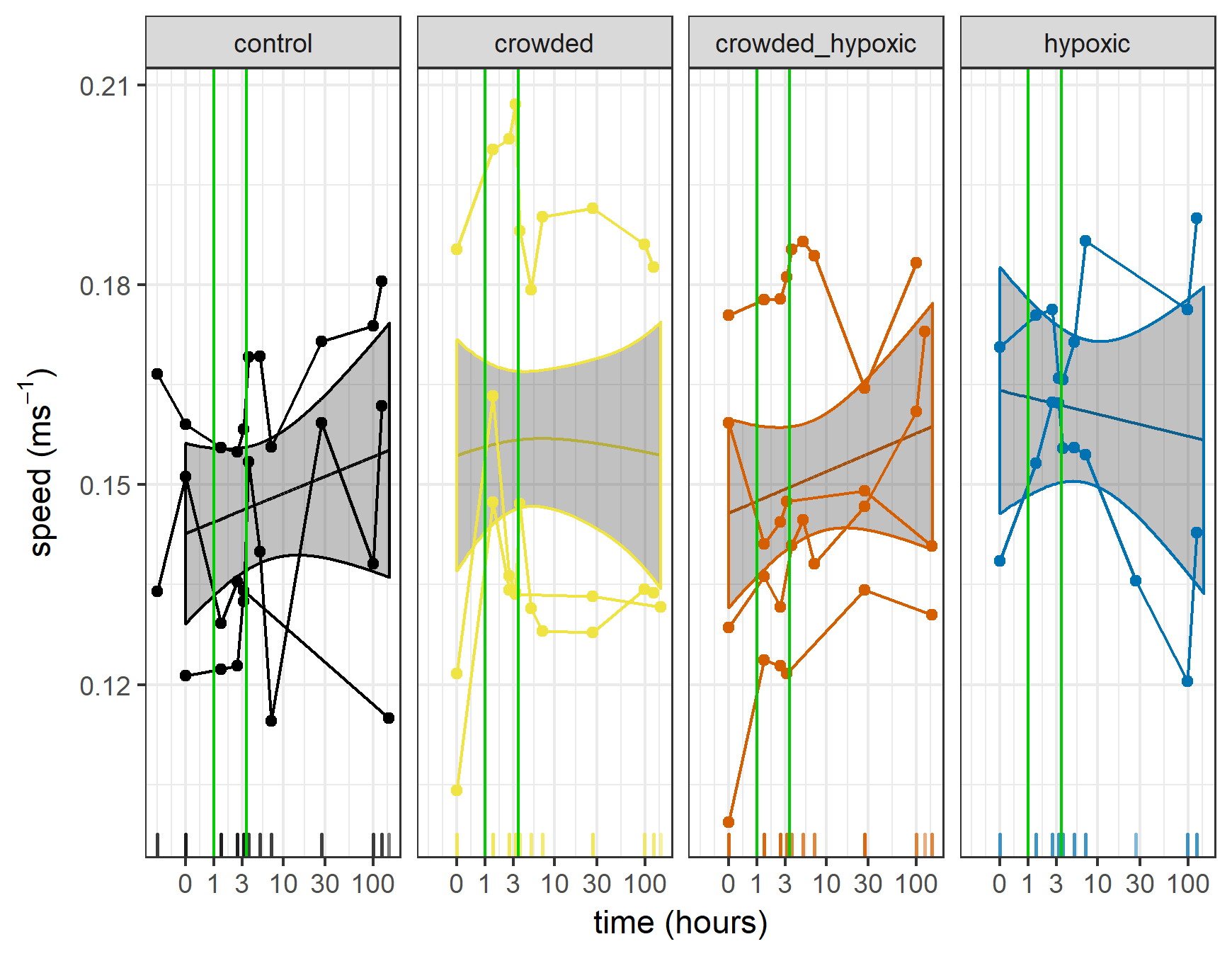

Supplement: S1 Fig — (TIF) [file pone.0190259.s002.tif]
